# Correction: Strategic design of GalNAc-helical peptide ligands for efficient liver targeting

**DOI:** 10.1039/d5sc90027a

**Published:** 2025-01-24

**Authors:** Takahito Ito, Nobumichi Ohoka, Michihiko Aoyama, Takashi Nishikaze, Takashi Misawa, Takao Inoue, Akiko Ishii-Watabe, Yosuke Demizu

**Affiliations:** a Division of Organic Chemistry, National Institute of Health Sciences 3-25-26 Tonomachi Kawasaki Kanagawa 210-9501 Japan demizu@nihs.go.jp; b Graduate School of Medical Life Science, Yokohama City University 1-7-29 Yokohama Kanagawa 230-0045 Japan; c Division of Molecular Target and Gene Therapy Products, National Institute of Health Sciences Kanagawa Japan; d Division of Biological Chemistry and Biologicals, National Institute of Health Sciences 3-25-26 Tonomachi Kawasaki-ku Kawasaki Kanagawa 210-9501 Japan; e Solutions COE, Analytical & Measuring Instruments Division, Shimadzu Corporation 1 Nishinokyo Kuwabara-cho, Nakagyo-ku Kyoto 604-8511 Japan; f Graduate School of Medicine, Dentistry and Pharmaceutical Sciences, Division of Pharmaceutical Science of Okayama University 1-1-1 Tsushimanaka Kita 700-8530 Japan

## Abstract

Correction for ‘Strategic design of GalNAc-helical peptide ligands for efficient liver targeting’ by Takahito Ito *et al.*, *Chem. Sci.*, 2024, **15**, 18789–18795, https://doi.org/10.1039/D4SC05606J.

The authors regret that the unit of measurement of the IC_50_ values in Fig. 6 was incorrectly given. The corrected version of Fig. 6 and its caption can be found below.

**Fig. 1:**
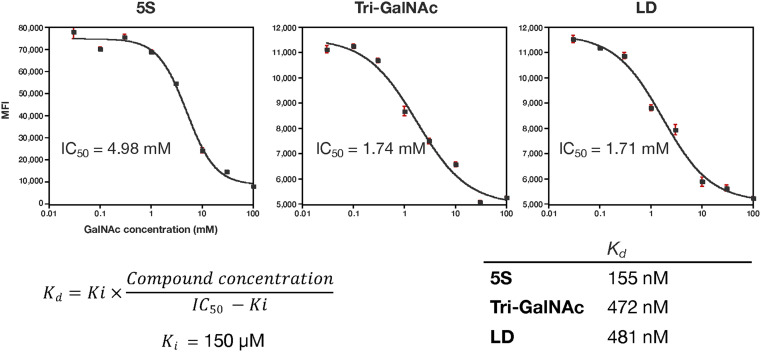
Cell-based binding study. HepG2 cells were treated with 5 mM compound in the presence of 0.03–100 mM GalNAc for 2 h. The mean fluorescence intensity (MFI) of three samples was recorded using a flow cytometer. The concentration of *N*-acetylgalactosamine (Gal-NAc) and MFI were plotted on the *X*- and *Y*-axis, respectively. Sigmoidal curves were fitted to plots to calculate the 50% inhibitory concentration (IC_50_) of GalNAc against the compounds tested. The dissociation constant (*K*_d_) was calculated by substituting the inhibition constant (*K*_i_; 150 mM) of GalNAc and the IC_50_ into the Cheng–Prusoff equation.

The authors also regret that the citation numbers in the final two paragraphs of the Results and discussion section and the Conclusions section were incorrect. The corrected version of these paragraphs, with correct citation numbering highlighted, and the relevant citations are given below.

Next, we evaluated the molecules’ delivery efficiency. The peptide was biotinylated and the uptake of streptavidin (54 kDa), which binds to the biotinylated ligands, was evaluated. We showed that peptide **5S-Bio** could transport approximately four times more streptavidin than Tri-GalNAc and significantly than streptavidin itself (SA) suggesting its potential to deliver large proteins that cannot pass through the cell membrane on their own (Fig. 7). Although, the uptake trends between peptide **5S-Bio** and **Tri-Bio** were similar to the result of FITC-labelled ligands, their differences became slightly smaller. Streptavidin forms a tetramer with biotin, which is able to bind to each streptavidin subunit. Therefore, there are four binding sites and the apparent binding activity of Tri-GalNAc may have increased due to this multivalent effect.^37^

Ligands comprising GalNAc or its analogs have been used as extracellular protein degraders (*e.g.*, lysosome-targeting chimeras [LYTACs] or ASGPR-targeting chimeras).^38–40^ LYTACs, which comprise a ligand that triggers endocytosis and a ligand that binds to the target protein, have recently attracted attention. Therefore, we attempted to use peptide **5S** as an endocytosis ligand for LYTACs. A conjugate compound comprising ligands and an anti-epidermal growth factor receptor (EGFR) antibody (cetuximab) was synthesized with an intended drug-to-antibody ratio of 8.0 (Fig. 8a). This ratio was subsequently confirmed to be 7.2 for **5S** and 7.4 for Tri-GalNAc *via* MALDI mass spectrometry. The EGFR degradation activity in HepG2 cells was evaluated using western blotting. Both peptide **5S** (**5S-ctx**) and Tri-GalNAc (**Tri-ctx**) conjugates showed significant degradation activity at 20 and 200 nM (Fig. 8b). Next, we evaluated the clearance of EGFR from membrane by flow cytometry. The experiment was conducted using 1 nM of antibody-conjugates on HepG2 cells. **5S-ctx** showed significant EGFR clearance at 8 h in HepG2 compared to **Tri-ctx**, and this clearance reached a plateau at 16 h (Fig. 8c). Although this experiment does not evaluate the degradation of EGFR, when combined with the western blot results, these findings suggest that **5S-ctx** may have a higher degree of EGFR degradation than **Tri-ctx**.

## Conclusions

In this study, we successfully developed peptide-based hepatocyte-targeting DDS ligands using helical structures to control the orientation of GalNAc moieties. Our key peptide, **5S**, demonstrated greater uptake efficiency and hepatocyte selectivity than the conventional Tri-GalNAc ligand. These findings underscore the effectiveness of our approach in enhancing ligand binding to ASGPR through geometric control. Our comprehensive analysis revealed that peptide **5S** could facilitate the endocytosis of large proteins, such as streptavidin, more efficiently than Tri-GalNAc. This indicates its potential applicability in delivering macromolecules that typically cannot penetrate cellular membranes by themselves. Furthermore, the use of peptide **5S** as an endocytosis ligand for LYTACs shows promise, despite the need for further optimization to enhance protein degradation efficiency. The flexibility of the GalNAc linker emerged as a critical factor in binding efficiency, highlighting the necessity for balanced structural rigidity and adaptability. Our molecular dynamics simulations and binding studies provided a deeper understanding of how the positional and structural attributes of GalNAc influence ASGPR engagement. Optimizing these ligands for *in vivo* applications remains essential. The inherent versatility of peptides, coupled with their ability to incorporate functional sequences, offers a promising avenue for overcoming current limitations.^41^ Overall, our approach involving the use of helical peptides to control ligand orientation represents a significant step forward in designing effective liver-targeted therapies. This methodology not only enhances the specificity and efficiency of DDS ligands but also holds potential for broader applications in targeting multivalent receptors, such as those involved in various viral infections.^42,43^ Our ongoing efforts aim to further refine these systems, potentially addressing membrane permeability challenges in drug discovery modalities that cannot penetrate membranes or target specific tissues themselves.^44–47^

37 S. Cecioni, A. Imberty and S. Vidal, Glycomimetics versus multivalent glycoconjugates for the design of high affinity lectin ligands, *Chem. Rev.*, 2015, **115**, 525–561.

38 G. Ahn, S. M. Banik, C. L. Miller, N. M. Riley, J. R. Cochran and C. R. Bertozzi, LYTACs that engage the asialoglycoprotein receptor for targeted protein degradation, *Nat. Chem. Biol.*, 2021, **17**, 937–946.

39 D. F. Caianiello, M. Zhang, J. D. Ray, R. A. Howell, J. C. Swartzel, E. M. J. Branham, E. Chirkin, V. R. Sabbasani, A. Z. Gong, D. M. McDonald, V. Muthusamy and D. A. Spiegel, Bifunctional small molecules that mediate the degradation of extracellular proteins, *Nat. Chem. Biol.*, 2021, **17**, 947–953.

40 Y. Zhou, P. Teng, N. T. Montgomery, X. Li and W. Tang, Development of Triantennary N-Acetylgalactosamine Conjugates as Degraders for Extracellular Proteins, *ACS Cent. Sci.*, 2021, **7**, 499–506.

41 T. Braulke and J. S. Bonifacino, Sorting of lysosomal proteins, *Biochim. Biophys. Acta*, 2009, **1793**, 605–614.

42 D. S. Kwon, G. Gregorio, N. Bitton, W. A. Hendrickson and D. R. Littman, DC-SIGN-Mediated Internalization of HIV Is Required for Trans-Enhancement of T Cell Infection, *Immunity*, 2002, **16**, 135–144.

43 B. Tassaneetrithep, T. H. Burgess, A. Granelli-Piperno, C. Trumpfheller, J. Finke, W. Sun, M. A. Eller, K. Pattanapanyasat, S. Sarasombath, D. L. Birx, R. M. Steinman, S. Schlesinger and M. A. Marovich, DC-SIGN (CD209) mediates dengue virus infection of human dendritic cells, *J. Exp. Med.*, 2003, **197**, 823–829.

44 H. Osawa, T. Kurohara, T. Ito, N. Shibata and Y. Demizu, CRBN ligand expansion for hematopoietic prostaglandin D(2) synthase (H-PGDS) targeting PROTAC design and their *in vitro* ADME profiles, *Bioorg. Med. Chem.*, 2023, **84**, 117259.

45 C. A. Foley, F. Potjewyd, K. N. Lamb, L. I. James and S. V. Frye, Assessing the Cell Permeability of Bivalent Chemical Degraders Using the Chloroalkane Penetration Assay, *ACS Chem. Biol.*, 2020, **15**, 290–295.

46 H. Yokoo, M. Naito and Y. Demizu, Investigating the cell permeability of proteolysis-targeting chimeras (PROTACs), *Expert Opin. Drug Discovery*, 2023, **18**, 357–361.

47 Y. Asami, K. Yoshioka, K. Nishina, T. Nagata and T. Yokota, Drug delivery system of therapeutic oligonucleotides, *J. Drug Discovery Ther.*, 2016, **19**, 256–262.

The Royal Society of Chemistry apologises for these errors and any consequent inconvenience to authors and readers.

